# Correlations between PNPLA3 Gene Polymorphisms and NAFLD in Type 2 Diabetic Patients

**DOI:** 10.3390/medicina57111249

**Published:** 2021-11-15

**Authors:** Oana Irina Gavril, Lidia Iuliana Arhire, Radu Sebastian Gavril, Madalina Ioana Zota, Andreea Gherasim, Otilia Nita, Andrei Drugescu, Andrei Catalin Oprescu, Irina Mihaela Esanu, Florin Mitu, Mariana Graur, Laura Mihalache

**Affiliations:** 1 Department of Medical Specialties (I), Faculty of Medicine, “Grigore T. Popa” University of Medicine and Pharmacy, 700115 Iași, Romania; ir.ungureanu@yahoo.com (O.I.G.); madalinachiorescu@gmail.com (M.I.Z.); andreidrugescu@yahoo.com (A.D.); esanu1925@gmail.com (I.M.E.); mitu.florin@yahoo.com (F.M.); 2 Department of Medical Specialties (II), Faculty of Medicine, “Grigore T. Popa” University of Medicine and Pharmacy, 700115 Iași, Romania; anny_gh2005@yahoo.com (A.G.); otilia.nita@umfiasi.ro (O.N.); graur.mariana@gmail.com (M.G.); laura_mvlad@yahoo.com (L.M.); 3 Morpho-Functional Department, Faculty of Medicine, “Grigore T. Popa” University of Medicine and Pharmacy, 700115 Iași, Romania; andreicatalinoprescu@yahoo.com

**Keywords:** hepatic steatosis, PNPLA3, insulin resistance, diabetes mellitus, cardiovascular risk

## Abstract

*Background and Objectives*: Non-alcoholic fatty liver disease is a worldwide significant public health problem, particularly in patients with type 2 diabetes mellitus. Identifying possible risk factors for the disease is mandatory for a better understandingand management of this condition. Patatin-like phospholipase domain-containing protein 3 (PNPLA3) has been linked to the development and evolution of fatty liver but not to insulin resistance. The aim of this study isto evaluate the relationships between PNPLA3 and fatty liver, metabolic syndrome and subclinical atherosclerosis. *Materials and Methods*: The study group consisted of patients with type 2 diabetes mellitus without insulin treatment. The degree of liver fat loading was assessed by ultrasonography, and subclinical atherosclerosis was assessed using carotid intima-media thickness (CIMT). PNPLA3 rs738409 genotype determination was performed by high-resolution melting analysis that allowed three standard genotypes: CC, CG, and GG. *Results*: Among the 92 patients, more than 90% showed various degrees of hepatic steatosis, almost 62% presented values over the normal limit for the CIMT. The majority of the included subjects met the criteria for metabolic syndrome. Genotyping of PNPLA3 in 68 patients showed that the difference between subjects without steatosis and subjects with hepatic steatosis was due to the higher frequency of genotype GG. The CC genotype was the most common in the group we studied and was significantly more frequent in the group of subjects with severe steatosis; the GG genotype was significantly more frequent in subjects with moderate steatosis; the frequency of the CG genotype was not significantly different among the groups.When we divided the group of subjects into two groups: those with no or mild steatosis and those with moderate or severe steatosis it was shown that the frequency of the GG genotype was significantly higher in the group of subjects with moderate or severe steatosis. PNPLA3 genotypes were not associated with metabolic syndrome, subclinical atherosclerosis, or insulin resistance. *Conclusions*: Our results suggest that PNPLA3 does not independently influence cardiovascular risk in patients with type 2 diabetes mellitus. The hypothesis that PNPLA3 may have a cardioprotective effect requires future confirmation.

## 1. Introduction

Nonalcoholic fatty liver disease (NAFLD) comprises a large spectrum of disorders from simple fat loading of the liver (hepatic steatosis—defined by a hepatocytic fat loading of at least 5%) to hepatic inflammation (NASH—nonalcoholic steatohepatitis), fibrosis, and cirrhosis, with its well-known complication, hepatocarcinoma [1]. While the global prevalence of this disorder is about 25% [2,3,4], the majority of patients with diabetes experience this condition [5]. The long-term evolution of patients with NAFLD has been intensely studied. Although only a small percentage of patients with hepatic steatosis will develop severe hepatic disorders, subjects with type 2 diabetes mellitus present an even higher risk of hepatocarcinoma [6]. Additionally, subjects with fatty liver have been shown to have a higher risk for surgical interventions and a higher rate of cancer in their first-degree relatives [7]. Previous studies have shown that patients with NAFLD have increased mortality compared to controls, with the most frequent cause of death being heart disease (28%) [8].

The hepatic accumulation of lipids seems to be an essential process in NAFLD physiopathology [9]. In 2008, a polymorphism of the PNPLA3 gene (patatin-like phospholipase domain-containing 3) was reported to be a determinant genetic factor of NAFLD [10]. PNPLA3, also called adiponutrin or calcium-independent phospholipase A2-epsilon, is part of a family of proteins with lipase/transacetylase activity [11]. PNPLA3 has been proved to play a significant role in determining the fatty hepatic load independent of obesity. Adiponutrin, a protein catalyzing the hydrolysis of triglycerides, is expressed specifically at the hepatocyte level in the fatty tissue and suprarenal glands [12]. Regarding the activity of PNPLA3 lipase on triglycerides and the activity of acylglycerol transacetylase, the gene expression is responsible for the lipase and for the accumulation of lipid droplets [13,14]. Moreover, it is greatly influenced by nutritional stimuli at the transcriptional and posttranscriptional levels [15]. Although several potential regulating mechanisms exist regarding the deposition of hepatic lipids, one of them may be the PNPLA3 gene, which affects the remodeling of triglycerides [16,17].

Clinical evidence supports the genetic transmission of NAFLD. PNPLA3 has been identified in children and adults who are sensitive to NAFLD and was associated with the severity of steatosis, acinar inflammation, hepatocyte bloating, and fibrosis [18].

The association between PNPLA3 and steatosis has been observed in seven out of eight genomic studies [19] and in many other studies that included Chinese and African American subjects, participants of the third National Health and Nutrition Examination Survey (NHANES) study [20], children with NAFLD and patients with morbid obesity [21,22]. The association between alleles of PNPLA3 and the histological severity of hepatic disease has also been confirmed. Recent studies have shown that alleles of PNPLA3 are associated with an increased risk of hepatocarcinoma. Several studies have identified the fact that a common variant of the PNPLA3 gene (allele G rs738409) is strongly associated with NAFLD susceptibility and the degree of hepatic steatosis [10,12,23]. Importantly, in patients with the PNPLA3 GG genotype, the association between allele rs738409 G and NAFLD is present only in subjects under 50 years old. However, these findings were seen in one study which included only 162 subjects [24].

NAFLD can be caused in an individual by the combination of obesity, insulinresistance, and genetic factors [25,26,27]. Although NAFLD traditionally leads to the progression of hepatic disease, the increase in mortality in these patients is due to cardiovascular disease. Moreover, in subjects with prediabetes, liver fat content has a stronger association with CIMT compared to visceral fat mass, independently of hyperglycemia and insulin resistance [28].

Genotyping for PNPLA3 could become part of the screening for patients with steatosis, as it might predict the risk for nonalcoholic steatohepatitis (NASH), hepatocellular carcinoma and cardiovascular disease. Cardiovascular risk depends on the components of metabolic syndrome, including NAFLD, but studies report divergent results related to worsening of this risk in patients with NAFLD [23] or have even suggested that some NAFLD genetic constellation could in fact provide cardioprotection [29].

In this study, the main interest is highlighting certain new relations between PNPLA3 genotypes and subclinical atherosclerosis for subjects with diabetes mellitus to confirm or invalidate the specificity of this gene for the stages of hepatic fatty disease as a marker or constituent of metabolic syndrome.

PNPLA3 increases the fatty load of the liver but was not yet proved to cause insulin resistance [30,31,32].The majority of patients with diabetes and obesity have ab initio insulinresistance and significant cardio-metabolic risk. Certain PNPLA3 alleles may be assumed to be cardioprotective in these patients.

The objective of this study isto assess the relation between PNPLA3 genotypes and the degree of hepatic fatty loading in subjects with type 2 diabetes mellitus, as well as with the degree of subclinical atherosclerosis and components of metabolic syndrome, and study the connection with the cardiovascular risk of this gene, viewed in the last few years as a marker of sensitivity regarding hepatic disease.

## 2. Materials and Methods

We performed an observational study on subjects with type 2 diabetes mellitus who did not receive insulin treatment, investigated in the Clinical Center for Diabetes, Nutrition and Metabolic Diseases of “Sf. Spiridon” Emergency Hospital Iași over a period of 18 months.The patients were evaluated in an outpatient—ambulatory setting. The inclusion criteria were as follows: type 2 diabetes mellitus treated with metformin and/or diet, subjects who signed the informed consent. The exclusion criteria were: subjects under insulin therapy, patients diagnosed with hepatitis B or C, toxic hepatitis, other hepatic conditions (Wilson’s disease), pathological alcohol consumption (more than two units a day for men and one unit for women).

Body weight was measured in the morning, fasting, barefoot using a calibrated scale. Waist circumference was also assessed in the morning, at the end of a normal expiration at the approximate midpoint between the lower margin of the iliac crest and the last rib.Blood samples were collected in the morning, fasting.

The degree of liver fatty loading was assessed by ultrasonography using a portable ultrasound *Carewell C12* with a convex probe of 3.5 MHz, and all subjects were assessed by the same examining physician.The followed parameters were liver to kidney contrast, parenchymal brightness, deep beam attenuation, bright vessel wall, and gallbladder wall definition [33].

Subclinical atherosclerosis was assessed using carotid intima-media thickness (CIMT) with a *Doppler Color LS 128* ultrasound, with a linear probe of 7.5 MHz. The ultrasonographic exam was performed using B mode for both common carotid arteries. CIMT higher than 1 mmwas considered abnormal, whereas values higher than 1.5mm were described as atheroma plaques [34].The measurements were assessed by the same examining physician.

We assessed the components of metabolic syndrome according to International Diabetes Federation (IDF) criteria [35]:

- Waist circumference (WC)—normal values for WC were <80 cm in women and <94 cm in men; values exceeding these limits led to the diagnosis of abdominal obesity;
- Blood pressure—previous diagnosis of hypertension or values higher than 130 mmHg systolic blood pressure or higher than 85 mmHg diastolic blood pressure at the time of the clinical examination;
- Hypertriglyceridemia (triglycerides >150 mg/dL);
- Low values of high-density lipoprotein cholesterol (HDLc) (<40 mg/dL in men, <50 mg/dL in women);
- Hyperglycemia or type 2 diabetes mellitus (all the subjects met this criteria).

The diagnosis of metabolic syndrome was made in the presence of three of the five disorders [35].

Insulin sensitivity was measured using homeostatic model assessment (HOMA-IR) using the following formula: HOMA-IR = (fasting glucose × insulinemia)/22.5 [36].

Genotyping of rs738409 was carried out by a high-resolution analysis of the dissociation of amplicons (HRM, high-resolution melting analysis) obtained by the amplification of a short genomic area that included the studied polymorphism. The HRM analysis was carried out with a Rotor-Gene 6000 instrument (Corbett Research, Australia).

For the purpose of obtaining amplicons containing the polymorphism site, two μL of genomic DNA extracted for standard genotyping was amplified with the help of 2 × SensiFastHRM master mix (Bioline, UK) and the primers GCCTTGGTATGTTCCTGCTTC and GGATAAGGCCACTGTAGAAGG were used at a final concentration of 200 nM.The thermal protocol applied was the activation of the enzyme for three minutes at 95 °C, followed by seven cycles of 10 s at 95 °C and 30 s at 67 °C, then 40 cycles of 10 s at 95 °C and 25 s at 60 °C. The length of the amplification process was 46 bp. The analysis of the dissociation curves was carried out using the device software (Rotor-Gene 6000 Series Software 1.7.87, Corbett Research, Australia).For the purpose of normalizing the curves, we selected a region of predissociation and one of postdissociation, inside which the relative fluorescence of each curve was considered to be 100% and 0%, respectively. The predissociation region was defined between 68.5 and 69.1 °C, while the postdissociation region was chosen between 77.0 and 78.6 °C.

**Figure 1:**
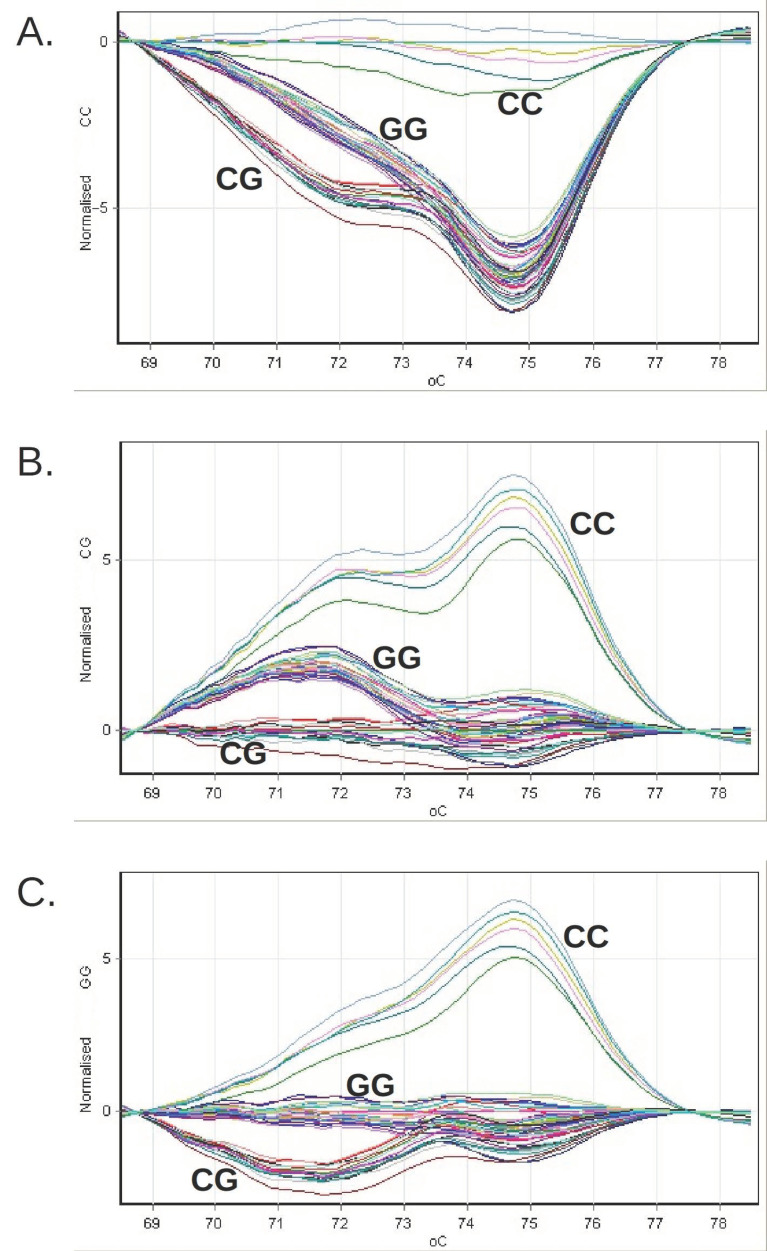
The grouping of the difference curves of the relative fluorescence obtained for the clinical genomic DNA samples during the genotyping by HRM or PNPLA3 on the Rotor-Gene 6000 system. The reference profiles were successively chosen, corresponding to the CC genotype (panel (**A**)), CG (panel (**B**)) and GG (panel (**C**)).

In parallel with the genomic DNA samples, we analyzed the following in each series of reactions (Figure 1):

- Three genotyping standards (CC, CG, GG), consisting of synthetic DNA molecules with a sequence that includes the genomic 46 bp region amplified with the help of the pair of primers used in the reaction;
- Three genotyping controls (rs738409 CC, CG, GG), consisting of genomic DNA sampled with a known PNPLA3 genotype; a negative amplification control, in which no DNA was introduced.

The statistical analysis was carried out using Statistica version 7.0 and SPSS v.20.The variables were described as mean ± standard deviation and with the 95%confidence interval for mean (if they were continuous variables) and as number and proportions (if they were discrete variables). When comparing average means between the two groups of continuous variables, the *t*-student test was performed (or the Mann–Whitney U test if the variances were not homogenous). For comparing more than two categories, for continuous variables, ANOVA test was performed (or Kruskal–Wallis test for non-homogenous variables). In ANOVA, if the statistical significance was obtained, post-hoc analysis was performed (Bonferroni test for homogenous variables or Tamhane test for non-homogenous variables) to identify the significant differences between categories. For comparingdiscrete variables, Chi square (χ^2^) was used (for χ^2^ the significance threshold was *p* = 0.1).We considered *p* < 0.05 indicative of statistical significance. Logistical regression was performed to identify independent predictors for NAFDL.

This research was conducted in accordance with the Declaration of Helsinki and had the ethical approval of the “Grigore T. Popa” University of Medicine and Pharmacy (no 17140, 3 August 2016). All participants signed informed consent before entering the study.

## 3. Results

Among the 92 patients (44 men, 48 women) with type 2 diabetes mellitus investigated, 68 (73.91%) were from an urban environment, and 24 subjects (26.09%) were from a rural environment. The average age of the group was 60.38 ± 10.37 years, varying from 33 to 86 years of age. The general characteristics of the study population are presented in Table 1 and Supplementary Table S1.

While hepatic fatty loading was absent in 9.8% of the subjects (9 patients), 26.1% of the cases presented mild steatosis (24 patients), 36.9% presented moderate steatosis (34 patients), and 27.2% presented severe steatosis (25 patients). HOMA-IR was found to be significantly higher in patients with severe steatosis, compared to all other categories and also CIMT was significantly higher in patients with moderate steatosis compared to those with mild steatosis (Supplementary Table S2). When dividing the patients in two groups, those with no or mild steatosis, and those with moderate or severe steatosis, we confirmed that both HOMA-IR and CIMT were significantly higher in those with moderate or severe steatosis (*p* < 0.001, *p* = 0.003 respectively). Moreover, metabolic syndrome was found in a significantly higher proportion in patients with moderate or severe steatosis (89.83% compared to 66.67%, *p* = 0.008) (Table 2).CIMT was above the normal limit (1mm) in 62% of the patients. Using binary logistic regression, we were able to show that steatosis associated with CIMT independently of age, sex, BMI, and WC (Table 3).

The majority of the included subjects (81.51%) met the criteria for metabolic syndrome (Table 1).

We performed the genetic analysis on a subgroup of 68 patients, which maintained the same general characteristics as the study population (Supplementary Table S3). The CC genotype was the most common in the group we studied, with no statistical differences between men and women (*p* = 0.297) (Table 4) and was significantly more frequent in the group of subjects with severe steatosis (73.68% compared to 48% in those with moderate steatosis; *p* = 0.04) (Table 5); the GG genotype was significantly more frequent in subjects with moderate steatosis (28% compared to 5.26% in those with severe steatosis; *p* = 0.03); the frequency of the CG genotype was not significantly different among the groups (*p* > 0.05).

As the frequency of the genotypes was the same in the group of subjects without steatosis and the group of subjects with mild steatosis, we combined the two groups and compared them with the groups of subjects with moderate and severe steatosis.

This method of comparison showed that the frequency of the CC genotype was significantly higher in the group of subjects with severe steatosis (73.68% compared to 48% in moderate steatosis; *p* = 0.04); the frequency of the CG genotype was significantly higher in subjects with no or mild steatosis (50% compared to 24% in subjects with moderate steatosis; *p* = 0.03 and 21.05% in subjects with severe steatosis; *p* = 0.03); the frequency of the GG genotype was significantly higher in subjects with moderate steatosis (28% versus 0% in subjects with normal liver or mild steatosis; *p* = 0.004).

Furthermore, we divided the group of subjects into two groups: those withno or mild steatosis and those with moderate or severe steatosis.

The frequency of the GG genotype was significantly higher in the group of subjects with moderate or severe steatosis (18.18% in comparison with 0% in the group of subjects with no or mild steatosis; *p* = 0.01) (Table 6).

No significant differences were found in terms of the PNPLA3 genotype regarding the degree of subclinical atherosclerosis, the presenceor the components of the metabolic syndrome, or HOMA-IR index (Table 7 and Supplementary Table S4).

## 4. Discussion

In this group of patients with diabetes, the prevalence of hepatic steatosis was very high (approximately 90%), and these results were similar to other recent data (a percentage varying from 50 to 90%) [6,37,38,39]. The fact that the prevalence of steatosis obtained inour research is at the upper limit of the results of other studies could be explained by the foodhabits specific to our region (high saturatedfats) aggravated by a sedentary lifestyle (especially for those in the urban environment).

The GG genotype was associated with an increase in the hepatic fat content. In this population of subjects with diabetes, the results confirmed the influence of the PNPLA3 polymorphism on the hepatic triglyceride content. An important finding of our research was the lack of statistically significant associations between the PNPLA3 genotypes and the components of metabolic syndrome. In the subgroups resulting from PNPLA3 genotyping, the comparison of the average CIMT values indicated a statistically significant difference between the CC and CG genotypes (*p* = 0.01). The lack of associations with the components of the metabolic syndrome suggested that the presence of the G allele was not connected with metabolic disorders in subjects with type 2 diabetes mellitus. These data were in accord with other studies, which proved that in the general population, the PNPLA3 polymorphism was closely connected with the hepatic fatty content, independent of adiposity or insulinresistance. A study that included 330 subjects and used magnetic resonance spectroscopy as a diagnostic method proved that carriers of the G allele presented a significantly higher hepatic fat content compared to individuals homozygous for the C allele [31].This study suggested that adiponutrin could be a keyfactor in understanding the mechanisms involved in the differentiation of benign fatty liver and fatty liver with metabolic consequences. Nonetheless, the PNPLA3 gene could manifest itself better in the company of certain predisposing factors of hepatic injury (obesity, alcohol consumption, hepatic viruses) [40].

PNPLA3 does not affect the components of metabolic syndrome [41]. In our study, the prevalence of metabolic syndrome did not present differences regarding the PNPLA3 genotypes, which indicated that the management of metabolic factors was important regardless of genotype.

We can consider the PNPLA3 genotype to be an important predictor of the degree of hepatic fat loading, even in subjects without metabolic syndrome. Hepatic steatosis can be viewed as a new component or even as a cause of this syndrome [42]. Nonetheless, the reason for which certain subjects without a metabolic risk develop hepatic steatosis remains incompletely clarified.

Although our study did not prove positive correlations between the presence of the GG genotype and subclinical atherosclerosis (increased CIMT), other authors reported contradictory results but only in subjects younger than 50 years of age [24]. A possible explanation is thatin young subjects who are not yet exposed to increased age-related cardiovascular risk, PNPLA3 can manifest its atherogenic role better.Moreover, the different types of fat distribution may impact on the relationship of the genotypes with hepatic steatosis and CIMT [43].This theory justifies the lack of a direct correlation between the PNPLA3 polymorphism and subclinical atherosclerosis in our study, as our study group consisted mainly of elderly people with a variety of associated cardiovascular risk factors (type 2 diabetes mellitus, obesity, dyslipidemia).One of the major limitations of our study is the limited number of subjects and the lack of the healthy control group.

## 5. Conclusions

Although the GG genotype presented a higher frequency in diabetic subjects with high hepatic fatty loading, it was not significantly correlated with subclinical atherosclerosis, insulin resistance (HOMA-IR), or elements of metabolic syndrome.

Our results suggest that PNPLA3 does not independently influence cardiovascular risk in patients with type 2 diabetes mellitus. The hypothesis that PNPLA3 may have a cardioprotective effect requires future confirmation.

## Figures and Tables

**Table 1:** General characteristics of the study population.

Parameters		Men (*n* = 44)	Women (*n* = 48)	Total (*n* = 92)	*p* Value *
Age (years) [Mean ± SD; 95% CI]		58.16 ± 11.04; 54.8–61.52	62.38 ± 9.27; 59.68–65.07	60.36 ± 10.32; 58.22–62.5	0.05
BMI (kg/m^2^) [Mean ± SD; 95% CI]		30.43 ± 5.08; 28.89–31.97	32.66 ± 5.49; 31.07–34.26	31.59 ± 5.39; 30.48–32.71	0.046
WC (cm) [Mean ± SD; 95% CI]		104.88 ± 11.33; 101.4–108.37	104.52 ± 12.56; 100.79–108.25	104.7 ± 11.92; 102.19–107.21	0.887
HOMA-IR [Mean ± SD; 95% CI]		6.33 ± 3.938; 5.036–7.625	6.549 ± 3.708; 5.363–7.735	6.443 ± 3.799; 5.586–7.299	0.801
Metabolic syndrome present [*n*; %]		33; 75	42; 87.5	75; 81.52	0.064
CIMT (mm) [Mean ± SD; 95% CI]		0.999 ± 0.133; 0.958–1.04	1.001 ± 0.152; 0.957–1.045	1 ± 0.143; 0.97–1.03	0.942
Degree of steatosis	none [*n*; %]	5; 11.37	4; 8.33	9; 9.78	0.703
	mild [*n*; %]	9; 20.45	15; 31.25	24; 26.1	
	moderate [*n*; %]	17; 38.64	17; 35.42	34; 36.96	
	severe [*n*; %]	13; 29. 54	12; 25	25; 27.17	

* Difference between men and women; SD = standard deviation; CI = confidence interval for mean; BMI = body mass index; WC = waist circumference; HOMA-IR = homeostatic model assessment for insulin resistance; CIMT = carotid intima-media thickness.

**Table 2:** Characteristics of the study population in relation to the presence of moderate or severe steatosis.

Parameters	No Steatosis or Mild Steatosis (*n* = 33)	Moderate or Severe Steatosis (*n* = 59)	*p* Value *
Age (years) [Mean ± SD; 95% CI]	59.85 ± 8.72; 56.76–62.94	60.41 ± 11.13; 57.49–63.34	0.802
BMI (kg/m^2^) [Mean ± SD; 95% CI]	32.2 ± 4.35; 30.66–33.74	31.26 ± 5.95; 29.69–32.82	0.151^#^
WC (cm) [Mean ± SD; 95% CI]	104.97 ± 10.48; 101.12–108.81	104.58 ± 12.81; 101.18–107.98	0.885
HOMA-IR [Mean ± SD; 95% CI]	4.21 ± 2.454; 3.219–5.201	7.559 ± 3.877; 6.48–8.638	<0.001
Metabolic syndrome present [*n*; %]	22; 66.67	53; 89.83	0.008
CIMT (mm) [Mean ± SD; 95% CI]	0.939 ± 0.109; 0.91–0.978	1.034 ± 0.149; 0.995–1.074	0.003 ^#^

* Difference between patients with no or mild steatosis and patients with moderate or severe steatosis; ^#^ using Mann–Whitney U non-parametric test; SD = standard deviation; CI = confidence interval for mean; BMI = body mass index; WC = waist circumference; HOMA-IR = homeostatic model assessment for insulin resistance; CIMT = carotid intima-media thickness.

**Table 3:** Correlations between CIMT and hepatic steatosis.

Parameters	Unadjusted		Adjusted	
	OR (95% CI)	*p*	OR (95% CI)	*p*
Presence of moderate or severe steatosis	3.23 (1.21–8.63)	0.019	3.06 (1.11–8.45)	0.031
Age (years)	1.02 (0.98–1.07)	0.303	1.02 (0.97–1.07)	0.342
Sex	0.82 (0.35–1.92)	0.644	1.003 (0.36–2.76)	0.995
BMI (kg/m^2^)	1.03 (0.95–1.11)	0.508	1.06 (0.886–1.27)	0.53
WC (cm)	1.005 (0.97–1.042)	0.776	0.99 (0.91–1.07)	0.736

**Table 4:** Distribution of the PNPLA3 genotypes in the group studied.

Genotype	Men (*n* = 28)	Women (*n* = 40)	Total (*n* = 68)
CC [*n*; %]	13; 46.4	25; 62.5	38; 55.9
CG [*n*; %]	12; 42.9	10; 25.0	22; 32.4
GG [*n*; %]	3; 10.7	5; 12.5	8; 11.8

**Table 5:** Genotype frequency in subjects with different stages of hepatic steatosis.

Geno-Type	Degree of Hepatic Steatosis								Total		*p*
	No Steatosis		Mild Steatosis		Moderate Steatosis		Severe Steatosis				
	*n*	%	*n*	%	*n*	%	*n*	%	*n*	%	
**CC**	3	50	9	50	12	48	14	73.68	38	55.88	0.04
**CG**	3	50	9	50	6	24	4	21.05	22	32.35	>0.05
**GG**	0	0	0	0	7	28	1	5.26	8	11.76	0.03
Total	6	8.82	18	26.47	25	36.76	19	27.94	68	100	

**Table 6:** Genotype frequency in normal liver and hepatic steatosis subjects.

Genotype	No or Mild Steatosis		Moderate or Severe Steatosis		Total		*p*
	*n*	%	*n*	%	*n*	%	
**CC**	12	50	26	59,09	38	55.88	0.24
**CG**	12	50	10	22.73	22	32.35	0.01
**GG**	0	0	8	18.18	8	11.76	0.01
Total	24	35.29	44	64.71	68	100	

**Table 7:** Characteristics of the study subpopulation in relation to the genotype identified.

Parameters	CC (*n* = 38)	CG (*n*= 22)	GG (*n* = 8)	*p* Value *
Age (years) [Mean ± SD; 95% CI]	61.82 ± 9.85; 58.58–65.05	61.36 ± 7.68; 57.96–64.77	59.00 ± 10.74; 50.02–67.98	0.74
BMI (kg/m^2^) [Mean ± SD; 95% CI]	31.69 ± 5.44; 29.9–33.48	32.18 ± 4.85; 30.03–34.33	31.44 ± 4.91; 27.33–35.54	0.918
WC (cm) [Mean ± SD; 95% CI]	104.03 ± 12.68; 99.74–108.32	105.33 ± 10.98; 100.33–110.33	105.88 ± 11.02; 96.66–115.09	0.883
HOMA-IR [Mean ± SD; 95% CI]	6.89 ± 3.39; 5.69–8.1	6.28 ± 5.074; 3.83–8.73	6.72 ± 1.7; 5.3–8.14	0.859
Metabolic syndrome present [*n*; %]	32; 84.21	17; 77.27	8; 100	0.326
CIMT (mm) [Mean ± SD; 95% CI]	1.01 ± 0.15; 0.96–1.06	0.97 ± 0.12; 0.91–1.02	0.99 ± 0.14; 0.88–1.11	0.609

* Difference between the three genotypes; SD = standard deviation; CI = confidence interval for mean; BMI = body mass index; WC = waist circumference; HOMA-IR = homeostatic model assessment for insulin resistance; CIMT = carotid intima-media thickness.

## Data Availability

The data presented in this study are available on request from the corresponding author.

## Supplementary Materials

The following are available online at https://www.mdpi.com/article/10.3390/medicina57111249/s1. Table S1: In depth description of the study population, Table S2: a. Characteristics of the study population in relation to the degree of steatosis, b. Post Hoc Tests (Multiple Comparisons) for HOMA-IR and CIMT between thegroups with different degrees of steatosis, Table S3: General characteristics of the study subgroup with genetic testing, Table S4: a. In depth characteristics of the study subpopulation in relation to the genotype identified, b. Post Hoc Tests (Multiple Comparisons) for Total cholesterol and HDL-cholesterolbetween thegroups with different genotypes.
