# Supplementary material for: Correlations between PNPLA3 Gene Polymorphisms and NAFLD in Type 2 Diabetic Patients
Source: Medicina (Kaunas). 2021 Nov 15;57(11):1249. doi: 10.3390/medicina57111249 (PMC8620174; doi:10.3390/medicina57111249)
Supplement: Supplementary file 1 [file medicina-57-01249-s001.zip › medicina-1447800-supplementary.pdf]

Table S1. In depth description of the study population

	Men (n=44)	Women (n=48)	Total (n=92)	p value*
Urban residence [n; %]	34; 77.27	34; 70.83	68; 73.91	0.332
Weight (kg) [Mean±SD; 95% CI]	91.45±16.31; 86.49-96.41	84.91±16.31; 80.17-89.64	88.04±16.55; 84.61-91.47	0.058
Height (cm) [Mean±SD; 95% CI]	173.18±6.29; 171.27-175.09	161.06±7.16; 158.98-163.14	166.86±9.07; 164.98-168.74	<0.001
SBP (mmHg) [Mean±SD; 95% CI]	145±20.79; 138.52-151.48	138.91±21.62; 132.42-145.41	141.85±21.32; 137.31-146.39	0.185
DBP (mmHg) [Mean±SD; 95% CI]	86.83±10.92; 83.43-90.24	84.73±9.53; 81.87-87.60	85.75±10.22; 83.57-87.93	0.341
Glycemia (mg/dl) [Mean±SD; 95% CI]	141.44±32.85; 131.33-151.55	143.83±34.92; 133.69-153.97	142.7±33.79; 135.67-149.74	0.738
HbA1c (%) [Mean±SD; 95% CI]	6.99±1.34; 6.59-7.41	6.81±1.08; 6.49-7.12	6.9±1.21; 6.65-7.15	0.459
Total cholesterol (mg/dl) [Mean±SD; 95% CI]	203.91±46.58; 189.57-218.24	206.23±52.48; 190.99-221.47	205.13±49.52; 194.82-215.44	0.825
LDL-cholesterol (mg/dl) [Mean±SD; 95% CI]	133.80±41.56; 120.85-146.76	130.19±48.7; 115.56-144.82	131.94±45.17; 122.31-141.56	0.712
HDL-cholesterol (mg/dl) [Mean±SD; 95% CI]	40.21±9.41; 37.31-43.11	41.67±11.82; 38.23-45.1	184.44±137.01; 142.28-226.61	0.52
Triglycerides (mg/dl) [Mean±SD; 95% CI]	184.44±137.01; 142.28-226.61	162.71±73.3; 141.42-183.99	172.98±108.1; 150.46-195.49	0.341

*Difference between Men and Women; SD=standard deviation; CI=Confidence Interval for Mean; SBP=systolic blood pressure; DBP=diastolic blood pressure

Table S2.a. Characteristics of the study population in relation to the degree of steatosis

	No steatosis (n=9)	Mild steatosis (n=24)	Moderate steatosis (n=34)	Severe steatosis (n=25)	p value*
Age (years) [Mean±SD; 95% CI]	62.00±8.22; 55.68-68.32	59.04±8.93; 55.27-62.81	61.52±10.37; 57.84-65.19	58.96±12.12; 53.96-63.96	0.692
BMI (kg/m^2) [Mean±SD; 95% CI]	33.01±5.12; 29.08-36.94	31.9±4.1; 30.17-33.63	30.5±5.68; 28.49-32.51	32.26±6.26; 29.67-34.84	0.498
WC (cm) [Mean±SD; 95% CI]	106.75±12.75; 96.09-117.41	104.35±9.82; 100.1-108.59	103.59±12.16; 99.21-107.98	105.84±13.75; 100.16-111.52	0.865
HOMA-IR [Mean±SD; 95% CI]	3.329±1.51; 1.744-4.913	4.474±2.646; 3.236-5.713	6.404±3.612; 5.055-7.752	9.134±3.737; 7.477-10.791	<0.001
Metabolic syndrome present [n; %]	5; 55.55	17; 70.83	29; 85.29	24; 96	0.023
CIMT (mm) [Mean±SD; 95% CI]	0.894±0.131; 0.794-0.995	0.956±0.098; 0.915-0.998	1.039±0.128; 0.994-1.085	1.028±0.174; 0.956-1.03	0.014#

Table S2.b. Post Hoc Tests (Multiple Comparisons) for HOMA-IR and CIMT between the groups with different degrees of steatosis

Dependent Variable	(I) Degree of steatosis	(J) Degree of steatosis	Mean Difference (I-J)	Std. Error	Sig.	95% Confidence Interval	
						Lower Bound	Upper Bound
HOMA-IR Bonferroni test	**none**	mild	-1.14580	1.54576	1.000	-5.3364	3.0448
		moderate	-3.07490	1.48511	.251	-7.1011	.9513
		**severe**	-5.80551*	1.52946	**.002**	-9.9519	-1.6591
	**mild**	none	1.14580	1.54576	1.000	-3.0448	5.3364
		moderate	-1.92910	.95864	.287	-4.5280	.6698
		**severe**	-4.65972*	1.02599	**.000**	-7.4412	-1.8782
	**moderate**	none	3.07490	1.48511	.251	-.9513	7.1011
		mild	1.92910	.95864	.287	-.6698	4.5280
		**severe**	-2.73062*	.93213	**.027**	-5.2576	-.2036
	**severe**	**none**	5.80551*	1.52946	**.002**	1.6591	9.9519
		**mild**	4.65972*	1.02599	**.000**	1.8782	7.4412
		**moderate**	2.73062*	.93213	**.027**	.2036	5.2576
CIMT (mm) Tamhane test	none	mild	-.06181	.04803	.781	-.2138	.0902
		moderate	-.14495	.04906	.067	-.2978	.0079
		severe	-.13356	.05589	.154	-.2976	.0305
	**mild**	none	.06181	.04803	.781	-.0902	.2138
		**moderate**	-.08314*	.03004	**.045**	-.1651	-.0012
		severe	-.07175	.04023	.403	-.1834	.0399
	**moderate**	none	.14495	.04906	.067	-.0079	.2978
		**mild**	.08314*	.03004	**.045**	.0012	.1651
		severe	.01139	.04145	1.000	-.1030	.1258
	severe	none	.13356	.05589	.154	-.0305	.2976
		mild	.07175	.04023	.403	-.0399	.1834
		moderate	-.01139	.04145	1.000	-.1258	.1030

*Difference between the four degrees of steatosis; #using Kruskal-Wallis non-parametric test; SD=standard deviation; CI=Confidence Interval for Mean; BMI=body mass index; WC=waist circumference; HOMA-IR= homeostatic model assessment for insulin resistance; CIMT=carotid intima-media thickness

Table S3. General characteristics of the study subgroup with genetic testing

		Men (n=28)	Women (n=40)	Total (n=68)	p value*
Age (years) [Mean±SD; 95% CI]		60.93±9.54; 57.23-64.63	61.63±9.09; 58.72-64.53	61.34±9.21; 59.11-63.57	0.762
BMI (kg/m^2) [Mean±SD; 95% CI]		29.85±3.91; 28.33-31.37	33.19±5.47; 31.44-34.94	31.82±5.13; 30.57-33.06	0.007
WC (cm) [Mean±SD; 95% CI]		103.85±10.55; 99.68-108.02	105.26±12.74; 101.08-109.45	104.68±11.81; 101.75-107.60	0. 639
HOMA-IR [Mean±SD; 95% CI]		6.65±4.05; 5.01-8.29	6.7±3.67; 5.41-7.8	6.68±3.81; 5.69-7.66	0.961
Metabolic syndrome present [n; %]		22; 78.57	35; 87.5	57; 83.82	0.256
CIMT (mm) [Mean±SD; 95% CI]		0.98±0.14; 0.93-1.03	1±0.15; 0.95-1.05	0.99±0.14 0.96-1.03	0. 555
Degree of steatosis	none [n; %]	3; 10.71	3; 7.5	6; 8.82	0.81
	mild [n; %]	6; 21.43	12; 30	18; 26.47	
	moderate [n; %]	10; 35.71	15; 37.5	25; 36.76	
	severe [n; %]	9; 32.14	10; 25	19; 27.94	

*Difference between Men and Women; SD=standard deviation; CI=Confidence Interval for Mean; BMI=body mass index; WC=waist circumference; HOMA-IR= homeostatic model assessment for insulin resistance; CIMT=carotid intima-media thickness

Table S4.a. In depth characteristics of the study subpopulation in relation to the genotype identified

	CC (n=38)	CG (n=22)	GG (n=8)	p value*
Weight (kg) [Mean±SD; 95% CI]	86.49±14.6; 81.69-91.29	90.73±15.96; 83.65-97.81	89.00±18.95; 73.16-104.84	0.591
Height (cm) [Mean±SD; 95% CI]	165.58±8.78; 162.69-168.47	168.05±9.2; 163.97-172.12	167.88±8.93; 160.4-175.35	0. 543
SBP (mmHg) [Mean±SD; 95% CI]	144.06±18.61; 137.56-150.55	138.57±21.97; 128.57-148.57	141.88±22.79; 122.82-160.93	0. 625
DBP (mmHg) [Mean±SD; 95% CI]	87.21±9.55; 83.87-90.54	84.52±12.03; 79.05-90	87.88±7.45; 81.64-94.11	0. 585
Glycemia (mg/dl) [Mean±SD; 95% CI]	138.29±36.87; 126.17-150.41	143.55±34.37; 128.31-158.79	144±16.7; 130.04-157.96	0.815
HbA1c (%) [Mean±SD; 95% CI]	6.61±1.03; 6.27-6.95	7.01±1.18; 6.48-7.53	6.51±.564; 6.04-6.98	0.312
Total cholesterol (mg/dl) [Mean±SD; 95% CI]	205.61±46.38; 190.36-220.85	188.68±53.34; 165.03-212.33	243.38±54.24; 198.02-288.73	0.033
LDL-cholesterol (mg/dl) [Mean±SD; 95% CI]	128.68±44.77; 113.3-144.06	121.88±45.57; 101.68-142.09	168.38±50.66; 126.02-210.73	0.05
HDL-cholesterol (mg/dl) [Mean±SD; 95% CI]	39.13±9.33; 36.07-42.2	46.14±12.95; 40.39-51.88	38±11.98; 27.99-48.01	0.044
Triglycerides (mg/dl) [Mean±SD; 95% CI]	183.61±96.95; 151.74-215.47	135.77±55.95; 110.97-160.58	160.38±73.44; 98.98-221.77	0.107

*Difference between the three genotypes; SD=standard deviation; CI=Confidence Interval for Mean; SBP=systolic blood pressure; DBP=diastolic blood pressure

Table S4.b. Post Hoc Tests (Multiple Comparisons) for Total cholesterol and HDL-cholesterol between the groups with different genotypes

Dependent Variable	(I) PNPLA3 genotype	(J) PNPLA3 genotype	Mean Difference (I-J)	Std. Error	Sig.	95% Confidence Interval	
						Lower Bound	Upper Bound
Total cholesterol (mg/dl) Bonferroni test	CC	CG	16.923	13.289	.622	-15.73	49.58
		GG	-37.770	19.295	.164	-85.19	9.65
	**CG**	CC	-16.923	13.289	.622	-49.58	15.73
		**GG**	-54.693*	20.479	**.029**	-105.02	-4.36
	**GG**	CC	37.770	19.295	.164	-9.65	85.19
		**CG**	54.693*	20.479	**.029**	4.36	105.02
HDL-cholesterol (mg/dl) Bonferroni test	CC	CG	-7.005	2.924	.058	-14.19	.18
		GG	1.132	4.246	1.000	-9.30	11.57
	CG	CC	7.005	2.924	.058	-.18	14.19
		GG	8.136	4.507	.227	-2.94	19.21
	GG	CC	-1.132	4.246	1.000	-11.57	9.30
		CG	-8.136	4.507	.227	-19.21	2.94
